# Weight stigma and disordered eating behaviors during the COVID-19 pandemic: the mediating role of weight gain concern and psychological distress

**DOI:** 10.1007/s40519-023-01608-6

**Published:** 2023-09-27

**Authors:** Patricia Fortes Cavalcanti de Macêdo, Edleide Brito, Carla de Magalhães Cunha, Maria da Purificação Nazaré Araújo, Poliana Cardoso Martins, Mônica Leila Portela de Santana

**Affiliations:** 1 School of Nutrition, Federal University of Bahia, Campus Canela, Rua Basílio da Gama, Salvador, BA 40110-907 Brazil; 2 Department of Statistics, Federal University of Bahia, Salvador, Brazil

**Keywords:** Weight stigma, Disordered eating, Obesity, SEM

## Abstract

**Objectives:**

The purpose of this study is to investigate whether the relationship between weight stigma experiences and disordered eating behaviors during the COVID-19 pandemic is mediated by weight gain concern and psychological distress among university students with and without overweight.

**Methods:**

A cross-sectional study was conducted with university students from five regions of Brazil who participated in the baseline assessment of the Online Cohort on Eating Behavior and Health (July/August 2020). Information on the frequency of binge eating episodes, food restriction, and purging, as well as experienced weight stigma, weight gain concern, and psychological distress, were recorded in an online questionnaire. Stratified structural equation modeling (SEM) analyses were performed to address the research questions of interest.

**Results:**

Out of the total sample (*n* = 2511), 33.5% of participants reported experiencing weight stigma. The prevalence of binge eating episodes, food restriction, and purging was 43.7%, 24.1%, and 5.4%, respectively. These behaviors were more prevalent in individuals with overweight than in those without this condition. Furthermore, it was observed that weight gain concern and psychological distress mediated the relationship between weight stigma and disordered eating behaviors regardless of body weight status.

**Conclusions:**

Experiences of weight stigma and disordered eating behaviors were prevalent among Brazilian university students, especially among those with overweight. Weight gain concern and psychological distress appear to be important factors underlying the relationship between these constructs during the pandemic, and they can contribute to the development of targeted strategies for the prevention and management of disordered eating.

**Level of evidence:**

Level V, cross-sectional study.

**Supplementary Information:**

The online version contains supplementary material available at 10.1007/s40519-023-01608-6.

## Introduction

Since the beginning of the COVID-19 pandemic, body weight has been the focus of media and scientific coverage, with news highlighting obesity as a comorbidity that worsens viral infection and social isolation as a contributing factor to weight gain [1, 2]. Although this discussion has been grounded in health care considerations, it is inferred that this scenario may have contributed to the manifestation of weight stigma [1], predominantly directed toward individuals with higher body weight [3, 4].

Weight stigma is a multidimensional phenomenon that encompasses structural and individual forms of devaluation and derogation due to excess weight [5]. From this perspective, experiences of weight stigma, or experienced weight stigma, are situated at the individual level and focus on the perception of the individual who has undergone stigmatization-related experiences [6]. This construct is a well-known risk factor for the development of various disordered eating behaviors (DEB), or disordered eating (DE), in the pre-pandemic context [7–11].

Furthermore, it is worth considering that DEB encompasses various behaviors [12], ranging from binge eating to restrictive dieting for weight loss, which are prevalent among young adults and associated with higher body weight and body image dissatisfaction [13–15]. Although theoretical frameworks suggest that similar models can explain the effect of weight stigma on binge eating, restrictive eating, and purging behaviors [16, 17], understanding the associations between weight stigma and DEB during the pandemic is particularly relevant, because this context has been shown to be conducive to increased experiences of weight stigma [3, 18] and the development of DEB [19–21].

Beyond weight stigma, the literature highlights a multitude of variables that can directly or indirectly influence disordered eating behaviors (DEB). These variables encompass concerns related to appearance, body dissatisfaction, and the internalization of beauty ideals [22–25]. While classic theoretical models, such as the tripartite model, are commonly used to explain sociocultural factors in the development of DEB, the incorporation of weight stigma into models that encompass aspects like weight and appearance concerns is a relatively nascent endeavor [16, 17, 26]. Hence, the incorporation of sociocultural and psychological variables can aid in comprehending the intricate connections associated with the development of DEB, thereby expanding the existing literature.

In this regard, Rodgers et al. [27] propose that due to the disruption in routine during the pandemic, variables such as psychological distress and increased concern about weight and body shape may be associated with an increase in disordered eating behaviors. However, despite the inclusion of body dissatisfaction and mental health in previous models seeking to explain the relationship between weight stigma and DE [16, 17], specific weight gain concerns have not been considered thus far. In addition, understanding these relationships among university students is important given the high prevalence of DEB in this group [15, 23] and their increased vulnerability to poorer mental health outcomes [28].

Finally, the recognition of the implications of weight stigma on disordered eating behaviors is significant in both pre-pandemic and pandemic settings across various populations [29–32], but its assessment remains limited in Brazil. Thus far, two isolated studies have investigated the association between these constructs among Brazilian adolescents in the pre-pandemic period [33, 34], and one study was conducted with adults during the pandemic [35].

Therefore, considering that experiences of weight stigma are prevalent in Western countries [36], the fact that the pandemic intensifies these experiences [3, 4] and that this phenomenon may be associated with episodes of disordered eating behaviors in individuals with and without overweight [16, 17, 38], a broader understanding of these issues is important. Such clarifications are essential for planning prevention and intervention strategies for eating disorders.

**Fig. 1:**
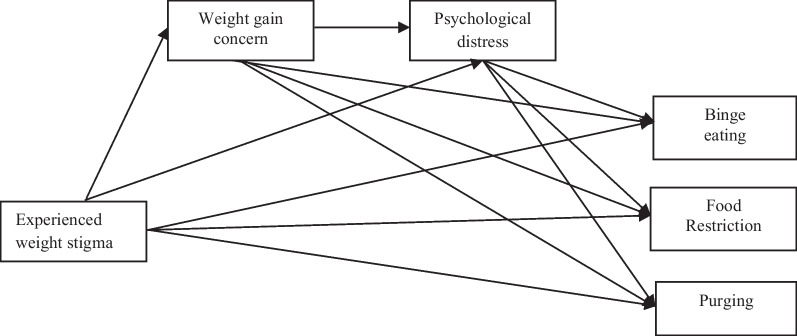
Conceptual model

The aim of this study is to test an adapted theoretical model based on O'Brien et al. (2016) [16] and Romano et al. (2020) [17]—incorporating the variable of weight gain concern—to clarify the relationship between experiences of weight stigma and disordered eating behaviors among Brazilian students, both those with and without overweight, during the COVID-19 pandemic. The underlying hypothesis is that this relationship is mediated by weight gain concern and psychological distress (see Fig. 1) in both weight groups during this period after controlling for confounding variables, such as sex, race/ethnicity, income, and age.

## Methodology

### Study design

This is a cross-sectional study using data from the Online Cohort on Eating Behavior and Health (COCASa: Cohort Online on Eating Behavior and Health). COCASa is a national longitudinal research project that aims to study the influence of the COVID-19 pandemic on eating behavior and mental health among students and faculty members from Brazilian universities. This cohort started in July 2020, and data collection was completed in October 2022. At the baseline of the study, a total of 3178 students and 1919 faculty members from public and private undergraduate institutions across the five regions of the country were included and responded to all questionnaires. This research was approved by the ethics committee of the School of Nutrition at the Federal University of Bahia (approval number 4,125,928) in accordance with national health regulations for research involving human subjects and the recommendations for conducting research during the pandemic. More detailed information about other methodological aspects and additional cross-sectional analyses of the COCASa study have been described in separate manuscripts with different objectives [35, 40].

### Participants and procedure

**Fig. 2:**
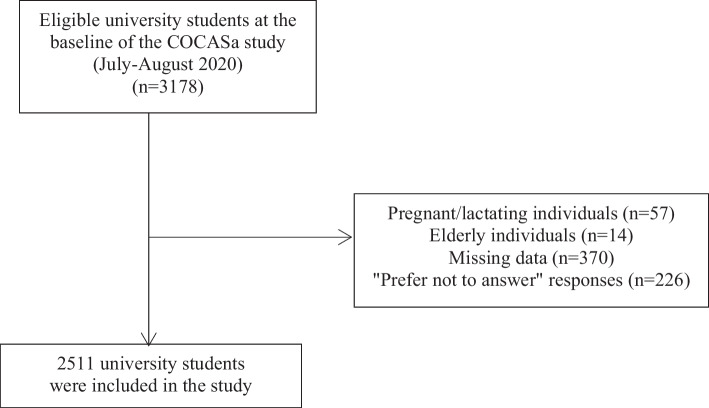
Flowchart illustrating participant selection

Participants included undergraduate students over the age of 18, of both sexes, enrolled in courses that had face-to-face activities suspended during the pandemic. Women who were pregnant or lactating and individuals over the age of 60 were not eligible to participate in this study. Out of the 3178 university students at baseline, the following exclusions were made: (1) 57 participants were excluded due to pregnancy/lactation (1.7%), (2) 14 participants were excluded due to being older adults (0.4%), and (3) after reviewing the questionnaires, 370 participants were excluded due to missing data (11.6%), and 226 participants were excluded because they selected the response option “prefer not to answer” for sociodemographic variables (7.1%). Thus, the final sample consisted of 2511 university students (1909 women and 602 men) (Fig. 2).

### Sample power

In this study, the sample power was calculated using power analysis techniques. For the mediation analysis, the method proposed by Sobel [41] was employed to estimate the confidence intervals for indirect effects in structural equation models. In addition, the pwrss package in R [42] was utilized to calculate the statistical power and minimum required sample size. This package provides a comprehensive set of tools for power analysis and sample size calculations across various statistical tests, including testing an indirect effect in mediation analysis. The calculations revealed a sample power of 90% considering the sample of 2511 university students, allowing for a robust examination of the relationships between experienced weight stigma, psychological distress, weight gain concern, and disordered eating behaviors.

### Measures

#### Disordered eating behaviors

Disordered eating behaviors were identified through five self-reported questions regarding weekly frequency in the past 3 months of binge eating, food restriction (eating little or fasting), and purging behaviors characterized by the use of diuretics, laxatives, and self-induced vomiting for weight loss. Participants responded to the questions using a 4-point Likert scale ranging from 1 = never per week to 4 = 2 or more times per week. These questions were proposed by Hay [43], adapted from the EDE-Q [40], validated for Brazilian adolescents [45], and previously used in studies with Brazilian university students [15, 25, 46, 47]. For each question, the response options were dichotomously scored (0 = No, 1 = Yes) according to the cutoff determined by Hay [43] for the presence of disordered eating behaviors (at least once a week). The variables for the use of diuretics, laxatives, or vomiting for weight loss were recategorized into a single dichotomous variable called purging behaviors, where positivity of the variable (Yes = 1) was considered for the occurrence of one or more of these symptoms weekly in the past 3 months (Supplementary Table S1). It is important to note that disordered eating behaviors were not assessed as a single construct but rather as three separate variables: binge eating, food restriction, and purging behaviors, following the approach adopted by other researchers [15, 25, 46, 47].

#### Experienced weight stigma

Experienced weight stigma was assessed through the question, “Have you ever been discriminated against or harassed because of your weight?’ (No; Yes, due to excess weight; Yes, due to low weight). Single-item measures have been used to track weight discrimination trends before [48, 49] and during the COVID-19 pandemic [32, 38, 50]. This measure reflects subjective experiences of weight stigma rather than objective experiences [51]. As the construct of interest in this study was discrimination due to excess weight, the data were regrouped into No (= 0; those who answered “no” or “yes, due to low weight”) and Yes (= 1; if the participant responded “yes, due to excess weight”) (Supplementary Table S1).

#### Weight gain concern during the pandemic

Weight gain concern was assessed using the question, “Has concern about weight gain become prominent in your life since the start of the COVID-19 pandemic?” The question was in a forced-choice format of “yes” or “no”.

#### Psychological distress

Psychological distress was evaluated using the 21 items of the Depression, Anxiety, and Stress Scale (DASS-21; [52]), which has demonstrated adequate validity and reliability, including in samples of Brazilian university students [53]. The DASS-21 consists of three subscales of 7 items each (depression, anxiety, and stress), scored on a Likert scale ranging from 0 (never) to 3 (almost always). Item scores within their respective subscales are summed, resulting in scores ranging from 0 to 21. In addition, a composite score of depression, anxiety, and stress, representing overall psychological distress (DASS-total), can be calculated (range: 0–63). In the current study, only the DASS-total score was examined. Higher scores indicate greater psychological distress (Cronbach’s alpha for this sample = 0.95).

#### Sociodemographic and anthropometric status variables

Participants self-reported their date of birth, gender, race/skin color, monthly income, weight (kg), and height (cm). Age was calculated by subtracting the date of questionnaire response from the date of birth. All sociodemographic variables were dichotomized (see Supplementary Table S1).

Body mass index (BMI) was calculated based on weight and height data using the formula BMI = kg/m^2^. Anthropometric status was determined using cutoff points for underweight (< 18.5 kg/m^2^), normal weight (between 18.5 kg/m^2^ and < 25 kg/m^2^), overweight (between 25 kg/m^2^ and < 30 kg/m^2^), and obesity (≥ 30 kg/m^2^) for adults and BMI-for-age percentiles: underweight (< 3rd percentile), normal weight (≥ 3rd percentile and < 85th percentile), overweight (≥ 85th percentile and < 97th percentile), and obesity (≥ 97th percentile) for 18–19-year-old university students [51]. For analysis purposes, participants were divided into two categories: without overweight (underweight and normal weight) and with overweight (overweight and obesity).

### Statistical analysis

Descriptive analyses were performed on all variables of interest, checking for normal distribution using the Kolmogorov‒Smirnov test and assessing homogeneity of variances using Levene’s test. Differences between participants without and with overweight in continuous variables were assessed using the Mann‒Whitney U test, given the nonnormality of the data, while the chi-square test was applied to categorical variables. A significant interaction was observed between anthropometric status and weight stigma regarding binge eating and purging, leading to stratified analyses for the two anthropometric status (without overweight and with overweight).

The relationship between experienced weight stigma, psychological distress, and disordered eating behaviors was investigated using structural equation modeling (SEM), with model fit assessed based on established criteria: root mean square error of approximation (RMSEA) ≤ 0.08, comparative fit index (CFI) > 0.95, Tucker‒Lewis index (TLI) > 0.90, and standardized root mean square residual (SRMR) < 0.08 [55, 56]. The robust method with the DWLS (diagonally weighted least squares) estimator was chosen to compute the standard errors of indirect effects. This method is particularly suitable for handling nonnormal variables and categorical or ordinal data. Complete case exclusion was adopted to handle missing data. The software packages R (version 3.6.3, 2020, Vienna, Austria: The R Foundation) and JASP (version 0.17.1, JASP Team University of Amsterdam, Amsterdam, The Netherlands) were used for the analyses.

## Results

### Sociodemographic variables of interest for the study according to anthropometric status

Out of the 2511 participants, 35.4% were classified as overweight. As described in Table 1, individuals with overweight had a higher median age (24.1; IQR = 8.7). The median BMI was 21.4 (IQR = 3.4) in the nonoverweight group and 28.2 (IQR = 4.7) in the group with overweight. Female gender, self-reported white race/ethnicity, and lower monthly family income were frequent in both studied groups. In the total sample, 33.5% of participants reported experiencing weight stigma, a frequency that was three times higher in individuals with overweight than in those without excess weight (Table 1).

**Table 1:** Distribution of variables of interest for the study stratified by anthropometric status

	Total (*N* = 2511)	Without overweight (*n* = 1623)	Overweight (*n* = 888)	p*
Characteristics				
Age, Md (IQR)	22.7 (5.8)	22.3 (4.6)	24.1 (8.7)	< 0.001
BMI, Md (IQR)	23.2 (6.0)	21.4 (3.4)	28.2 (4.7)	< 0.001
Sex, *n* (%)				
Female	1909 (76.0)	1282 (79.0)	627 (70.6)	< 0.001
Male	602 (24.0)	341 (21.0)	261 (29.4)	
Race/Color, *n* (%)				
White	1280 (51.0)	832 (51.3)	448 (50.5)	0.3
Non-White	1231 (49.0)	791 (48.7)	440 (49.5)	
Monthly family income, *n* (%)				
Less than or equal to R$ 3000.00	1433 (57.1)	946 (58.3)	487 (54.8)	0.05
Greater than or equal to R$ 3001.00	1078 (42.9)	677 (41.4)	401 (45.2)	
Weight stigma experienced, *n* (%)				
No	1670 (66.5)	1343 (82.7)	327 (36.8)	< 0.001
Yes	841 (33.5)	280 (17.3)	561 (63.2)	
Binge eating, *n* (%)				
No	1414 (56.3)	1022 (63.0)	392 (44.1)	< 0.001
Yes	1097 (43.7)	601 (37.0)	496 (55.9)	
Food restriction, *n* (%)				
No	1905 (75.9)	1308 (80.6)	597 (67.2)	< 0.001
Yes	606 (24.1)	315 (19.4)	291 (32.8)	
Purge behaviors, *n* (%)				
No	2376 (94.6)	1672 (96.9)	804 (90.5)	< 0.001
Yes	135 (5.4)	51 (3.1)	84 (9.5)	
Weight gain concern, *n* (%)				
No	1166 (46.4)	943 (58.1)	223 (25.1)	< 0.001
Yes	1345 (53.6)	680 (41.9)	665 (74.9)	
Psychological distress, Md (IQR)	21 (23)	21 (22)	22 (24)	< 0.001

*Man Whitney test for continuous variables and Chi square for categorical variables

Md: median; IQR: interquartile range

Regarding the prevalence of disordered eating behaviors (DEB) between the groups, individuals with overweight had higher frequencies of binge eating (55.9% vs. 37%, *p* < 0.001), food restriction (32.8% vs. 19.4%, *p* < 0.001), and purging behaviors (9.5% vs. 3.1%, *p* < 0.001) than those without overweight. The frequency of weight gain concern and levels of psychological distress were also higher in individuals with overweight. Furthermore, among individuals with overweight, the prevalence of binge eating, food restriction, and purging behaviors was significantly higher among those who reported experiencing weight stigma (respectively, 40%, 23.4%, 7.4%) compared to those who did not report such experiences (respectively, 15.9%, 9.3%, 2%). Conversely, in the group without overweight, the opposite pattern of these behaviors emerged, with lower prevalence rates among individuals who reported weight stigma experiences (respectively, 8.6%, 6.9%, 1.2%) compared to those who did not report such experiences (respectively, 28.4%, 12.5%, 2%).

### Structural model

The initial structural model showed a poor fit with the data. To improve the model fit, we made adjustments by removing direct paths from experienced weight stigma to food restriction and purging, as these paths were found to be nonsignificant. After the modifications, the model showed an acceptable fit with a CFI value of 0.96, TLI of 0.96, SRMR of 0.005, and RMSEA of 0.097 (90% CI: 0.086, 0.010).

**Fig. 3:**
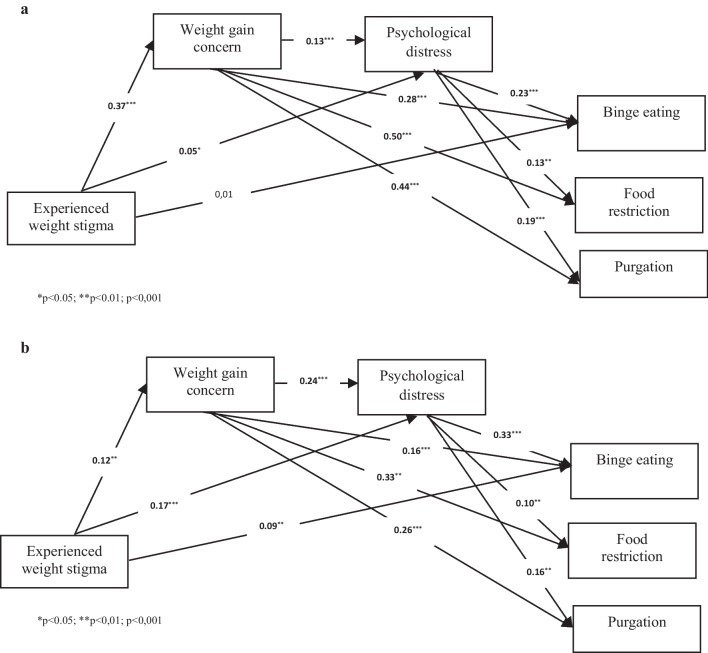
Structural model of standardized coefficients. **a** Structural model of the group without overweight; **b** Structural model of the group with overweight

In the analysis of the final model, controlling for age, sex, race/ethnicity, and income, it was observed that experienced weight stigma was directly associated with weight gain concern and higher levels of psychological distress in both individuals with and without overweight. In turn, these two constructs were directly associated with the three disordered eating behaviors: binge eating, food restriction, and purging. Figure 3 illustrates how weight gain concern and higher levels of psychological distress mediated the relationship between experienced weight stigma and disordered eating behaviors in both groups. Experienced weight stigma had a significant direct effect on binge eating only in individuals with overweight (B = 0.09, *p* < 0.01).

As demonstrated in Table 2, nearly all indirect effects connecting experiences of weight stigma to subsequent outcomes, including weight gain concern, psychological distress, and heightened levels of respective DEB, were found to be statistically significant. The exceptions were the pathways from weight stigma to psychological distress to food restriction and purging among individuals without overweight, as well as the pathway involving weight stigma, psychological distress, and purging in individuals with overweight. Importantly, regardless of anthropometric status, the path from weight stigma to weight gain concern, and subsequently to all three DEB, exhibited statistically significant effects.

**Table 2:** Indirect effects of experienced weight stigma in the structural models stratified by anthropometric status

Indirect effects	Without overweight (*n* = 1623)				Overweight (*n* = 888)			
	b (SE)	95%CI		*p*	b (SE)	95%CI		*p*
		LL	UL			LL	UL	
Weight stigma → Weight gain concern → Binge eating Food restriction Purgation	0.114 (0.016) 0.230 (0.020) 0.086(0.016)	0.084 0.191 0.054	0.145 0.269 0.118	< 0.001 < 0.001 < 0.001	0.023 (0.010) 0.046 (0.018) 0.034 (0.014)	0.003 0.011 0.008	0.044 0.081 0.061	0.026 0.010 0.011
Weight stigma → Psychological distress → Binge eating Food restriction Purgation	0.114 (0.007) 0.009 (0.005) 0.005 (0.003)	− 4.250 × 10^–4^ 6.907 × 10^–4^ − 5.621 × 10^–4^	0.028 0.018 0.011	< 0.001 0.069 0.076	0.061 (0.014) 0.018 (0.008) 0.013 (0.007)	0.033 0.003 -0.002	0.089 0.034 0.027	< .001 0.019 0.081
Weight stigma → Weight gain concern → Psychological distress → Binge eating Food restriction Purgation	0.113 (0.003) 0.008 (0.002) 0.005 (0.002)	0.006 0.004 0.002	0.019 0.013 0.008	< 0.001 < 0.001 0.0001	0.012 (0.005) 0.003 (0.002) 0.002 (0.001)	0.002 4.366 × 10^–5^ − 5.035 × 10^–4^	0.021 0.007 0.005	0.013 0.047 0.105

LL = lower limit, UL = upper limit, CI = confidence interval

## Discussion

Based on a previous literature review, the present study is the first to investigate the prevalence of weight stigma and its relationship with disordered eating behavior in a national sample of Brazilian university students using a structural equation model. In the present study, the prevalence of experienced weight stigma was 33.5%, and the prevalence of binge eating, food restriction, and purging behaviors was 43.7%, 24.1%, and 5.4%, respectively, all of which were significantly higher (*p* < 0.001) in individuals with overweight compared to those without this condition. Furthermore, weight gain concern and psychological distress mediated the relationship between experienced weight stigma and disordered eating behaviors in both groups.

Although studies on the prevalence of weight stigma in Brazil are limited [33, 34], the results of this research demonstrate that a notable percentage of university students have experienced weight discrimination in the country. Compared to the international scenario, the present research corroborates previous studies conducted with adults during the same period using a similar instrument, whether in the total sample [50] or when stratified by weight status [38]. Given the sociocultural model of weight stigma [57], it is worth considering that the high frequency of weight stigma may be related to the significant concern with the appearance characteristics of Brazil [58]. It is valid to note that it is not possible to attribute these results solely to the pandemic due to the absence of published data on weight stigma with this population before the COVID-19 outbreak.

Regarding the prevalence of disordered eating behaviors stratified by weight status, the percentages identified in this study were twice as high as those found in previous research using the same instrument among university students in national [15] and international [43] settings and similar to those found for food restriction and binge eating in nonclinical samples during the pandemic [19]. Evidence suggests that the high prevalence and susceptibility to disordered eating behaviors during the pandemic may be related to a set of psychological, social, and individual factors, such as difficulty in emotion regulation [19], high BMI, and exposure to previous experiences of weight stigma [59].

The results of this study provide initial evidence supporting the model that relates weight stigma to disordered eating behaviors during the first wave of the COVID-19 pandemic. It was found that experiences of weight stigma were associated with binge eating, food restriction, and purging behaviors, and these relationships were mediated by weight gain concern and psychological distress. These findings expand and reinforce the importance of body image and psychological factors in understanding disordered eating behaviors demonstrated in previous research [16, 17], highlighting the importance of including weight gain concern in theoretical models that explain the association between weight stigma and disordered eating.

Moreover, our findings carry practical implications that extend beyond the research realm. By integrating ‘weight gain concern’ and ‘psychological distress’ into theoretical frameworks, we deepen our comprehension of the complex interplay among weight stigma, mental well-being, and eating behaviors. In practical terms, these results hold several key implications. Firstly, they reinforced the importance of interventions aimed at mitigating ‘weight gain concern’ as potentially effective in the prevention and treatment of disordered eating behaviors, such interventions that increase body acceptance and develop healthy eating skills [60], for example. In addition, they underscore the need for systematic assessments of 'weight gain concern' and 'psychological distress' in both clinical and research settings. Such assessments can identify individuals at risk and enable the tailoring of therapeutic approaches to their unique needs. Our discussion emphasizes how these findings not only expand our theoretical knowledge but also offer the potential to inform more effective and nuanced practices when addressing the intricate dynamics between weight stigma, mental health, and disordered eating behaviors.

In addition, although the explanatory models are similar in both weight groups, weight stigma had a significant direct relationship with disordered eating behaviors only in individuals with higher body weight. In this regard, the data presented in this study may support the weight-based stigma/excess weight cyclical model (COBWEBS), which proposes that the impact of weight stigma on overeating, particularly in individuals with overweight, occurs as a response to the stress resulting from weight-related stigmatizing experiences [61].

There are important theoretical and methodological considerations to interpret the results. First, individuals with higher body weight are considered marginalized identities, and the effects of weight stigma on disordered eating behaviors are compounded by other consequences in their lives. In this sense, during the pandemic, in addition to dealing with the inherent changes in the context, individuals with obesity faced additional concerns such as the lack of adequate equipment and structures to assist them in case of illness [62].

Furthermore, since the experienced weight stigma in this study corresponded to retrospective data, the reported stigma experiences among individuals without overweight/obesity may be retrospective self-reports of previous overweight or obesity [36]. In addition, the relationship between weight stigma and disordered eating behaviors in individuals with overweight may have been underestimated due to the low variability of weight stigma experiences among the participants [63]. It is also important to acknowledge that while the RMSEA value did not meet the predefined criteria for adequacy, it is well-established in the literature that model adequacy should be assessed by considering a combination of fit indices [64]. This underscores the importance of evaluating the overall fit of the structural model, recognizing that the RMSEA, being sensitive to deviations from normality in the data [65], may indicate less favorable fit outcomes in such cases. Therefore, caution should be exercised in interpreting the results.

Finally, stratifying the sample by weight status contributed to demonstrating that the frequencies of the outcomes according to weight stigma experiences differed between individuals with and without overweight, which may suggest that while the relationships between variables can be explained by similar models, the distributions manifest themselves differently among the groups and need to be understood in their singularities.

### Strengths and limitations

The strengths of the study include the use of a large sample of university students, which allowed for robust statistical analysis controlled for sociodemographic variables. The use of a population sample, rather than clinical samples, enables the evaluation of factors associated with disordered eating behaviors in a broader group of healthy individuals. Furthermore, students are a population susceptible to poorer mental health indicators, especially during the pandemic, which reinforces the importance of understanding the implications of psychological and sociocultural factors on disordered eating behaviors in this pandemic scenario. The relationship between constructs in the field of nutrition and mental health contributes to supporting the importance of multidisciplinary healthcare teams in the care of this population, considering the challenge of implementing strategies that synergistically address obesity and disordered eating behaviors in low-income countries [66].

Despite important contributions to the field of disordered eating, our study has some limitations. First, all stages of the study were conducted online due to pandemic restrictions, which may introduce information bias. Second, experiences of weight stigma were assessed retrospectively, which increases the possibility of recall bias. Third, weight stigma was assessed using dichotomous (yes/no) questions. Although brief tools are commonly used in epidemiological studies in the field, this type of item can lead to measurement errors. Finally, the cross-sectional nature of the study limits the inference of causal relationships between variables.

This study has several implications for future research. The high prevalence of experiences of weight stigma and its association with disordered eating behaviors in Brazil signals the need to pay attention to this indicator as a factor associated with such outcomes. In this regard, further research is needed to examine the pathways through which weight discrimination impacts disordered eating behaviors, as the assessment of mediators in this process can help identify individuals who are more vulnerable to the effects of weight stigma on eating behavior. In addition, conducting longitudinal studies can provide evidence about the direction of the association and assist in the development of programs aimed at the prevention and management of disordered eating behaviors in individuals with or without weigh excess.

### What is already known about this subject?

High frequencies of experienced weight stigma and disordered eating behaviors have been observed among university students, as well as the association between these variables. During the pandemic, few studies have assessed the relationship between weight stigma and disordered eating behaviors, and no studies on this topic in the Brazilian context have been conducted.

### What does this study add?

This is the only epidemiological study that provided the prevalence of experienced weight stigma according to weight status in a national sample of Brazilian university students during the COVID-19 pandemic. The study found a high prevalence of experiences of weight stigma, and all investigated disordered eating behaviors during the pandemic (binge eating, food restriction, and purging behaviors), which were notably higher in individuals with overweight. The relationship between the variables was mediated by weight gain concern and psychological distress, highlighting the importance of considering these constructs in the targeted development of prevention and control strategies for disordered eating behaviors in the future.

## Conclusion

The present study identified a high prevalence of experienced weight stigma and disordered eating behaviors in the early stages of the pandemic, particularly in individuals with overweight. As hypothesized, weight gain concern and psychological distress mediated the relationship between experiences of weight stigma and disordered eating behaviors during the first wave of the COVID-19 pandemic. The identification of these issues can be useful for the development of targeted strategies for the prevention and management of disordered eating behaviors.

### Supplementary Information

Below is the link to the electronic supplementary material.

Supplementary file1 (DOCX 18 KB)

## Data Availability

The datasets generated and/or analyzed during the current study are available upon request from the corresponding author.
