# Supplementary material for: Weight stigma and disordered eating behaviors during the COVID-19 pandemic: the mediating role of weight gain concern and psychological distress
Source: Eat Weight Disord. 2023 Sep 27;28(1):78. doi: 10.1007/s40519-023-01608-6 (PMC10533574; doi:10.1007/s40519-023-01608-6)
Supplement: Supplementary file 1 — Supplementary file1 (DOCX 18 KB) [file 40519_2023_1608_MOESM1_ESM.docx]

**Article title**: Weight stigma and disordered eating during the COVID-19 pandemic: The mediating role of weight gain concern and psychological distress

**Journal name**: Eating and Weight Disorders - Studies on Anorexia, Bulimia and Obesity

**Author names**: Patricia Fortes Cavalcanti de Macêdo^1^ Edileide Brito^2^ Carla de Magalhães Cunha^1^ Maria da Purificação Nazaré Araújo^1^ Poliana Cardoso Martins^1^ Mônica Leila Portela de Santana^1^

**Affiliation**: 1. Federal University of Bahia. School of Nutrition 2. Federal University of Bahia, Department of Statistics.

**Address of corresponding author and e-mail address**: School of Nutrition, Federal University of Bahia, Campus Canela, Rua Basílio da Gama, ZIP code: 40.110-907, Salvador, BA, Brazil. E-mail: macedopatriciafortes@gmail.com

| **Supplementary table 1**. Dichotomization of variables | | |
| --- | --- | --- |
| Variables | Coding of COCASa research | Dichotomization |
| Binge eating | Never = 1  Less than 1 time per week = 2  1 time per week = 3  2 or more times a week = 4 | No = 0 (never; less than 1 time per week)  Yes = 1 (1 time per week; 2 or more times per week) |
| Food restriction | Never = 1  Less than 1 time per week = 2  1 time per week = 3  2 or more times a week = 4 | No = 0 (never; less than 1 time per week)  Yes = 1 (1 time per week; 2 or more times per week) |
| Purgation | Computed by the sum of the dichotomized variables laxative, diuretic and vomiting. After the sum, the variable was recategorized. *When the sum >* 1, the variable was recoded with a value =1. When = 0, the value was maintained. | No purging = 0 (never; less than 1 time per week)  With purging = 1 (1 time per week; 2 or more times per week) |
| Stigma of weight experienced | No = 1  Yes, by excess weight = 2  Yes, by low weight = 3 | No = 0  Yes = 1 (yes, for being overweight; yes, for underweight) |
| BMI | Low weight = 1;  Eutrophic = 2;  Overweight = 3  Obesity = 4. | No overweight = 0 (underweight; eutrophic)  Overweight = 1 (overweight; obesity) |
| Sex | Female = 1  Male = 2  *I'd rather not answer = 3 | Male = 0  Female = 1 |
| Race/Color | Yellow = 1;  White = 2;  Indigenous = 3;  Brown = 4;  Black = 5;  *I'd rather not reply = 6 | Non-white = 0 (Yellow; Indigenous; Brown; Black)  White = 1 |
| Monthly family income | Less than $1200 = 1  Between R$ 1200 and R$ 3.000,00 = 2  Between R$ 3.001,00 to R$ 10.000 = 3  Greater than $10,001= 4  *I'd rather not reply = 5 | Monthly income greater than R$ 3,001.00 = 0 (Between R$ 3,001.00 and R$ 10,000; Greater than R$10,001)  Income less than R$ 3000 = 1 (Less than R$ 1200; Between R$ 1200 and R$ 3.000,00) |
| * Participants who answered "I'd rather not answer" were excluded. Thus, the item was not included in the dichotomous variable. | | |
